# Patterns of Gene Expression Associated with *Pten* Deficiency in the Developing Inner Ear

**DOI:** 10.1371/journal.pone.0097544

**Published:** 2014-06-03

**Authors:** Hyung Jin Kim, Jihee Ryu, Hae-Mi Woo, Samuel Sunghwan Cho, Min Kyung Sung, Sang Cheol Kim, Mi-Hyun Park, Taesung Park, Soo Kyung Koo

**Affiliations:** 1 Division of Intractable Diseases, Center for Biomedical Sciences, National Institute of Health, Chungcheongbuk-do, South Korea; 2 Interdisciplinary Program in Bioinformatics, Seoul National University, Seoul, South Korea; 3 Department of Statistics, Seoul National University, Seoul, South Korea; 4 Korean BioInformation Center (KOBIC), Korea Research Institute of Bioscience and Biotechnology, Daejeon, South Korea; Universitat Pompeu Fabra, Spain

## Abstract

In inner ear development, phosphatase and tensin homolog (PTEN) is necessary for neuronal maintenance, such as neuronal survival and accurate nerve innervations of hair cells. We previously reported that *Pten* conditional knockout (cKO) mice exhibited disorganized fasciculus with neuronal apoptosis in spiral ganglion neurons (SGNs). To better understand the genes and signaling networks related to auditory neuron maintenance, we compared the profiles of differentially expressed genes (DEGs) using microarray analysis of the inner ear in E14.5 *Pten* cKO and wild-type mice. We identified 46 statistically significant transcripts using significance analysis of microarrays, with the false-discovery rate set at 0%. Among the DEGs, expression levels of candidate genes and expression domains were validated by quantitative real-time RT-PCR and *in situ* hybridization, respectively. Ingenuity pathway analysis using DEGs identified significant signaling networks associated with apoptosis, cellular movement, and axon guidance (i.e., secreted phosphoprotein 1 (*Spp1*)-mediated cellular movement and regulator of G-protein signaling 4 (*Rgs4*)-mediated axon guidance). This result was consistent with the phenotypic defects of SGNs in *Pten* cKO mice (e.g., neuronal apoptosis, abnormal migration, and irregular nerve fiber patterns of SGNs). From this study, we suggest two key regulatory signaling networks mediated by *Spp1* and *Rgs4*, which may play potential roles in neuronal differentiation of developing auditory neurons.

## Introduction

The inner ear is derived from a simple patch of otic placode adjacent to the hind brain. After formation of the otic cup and vesicle, otic neuroblasts delaminate from the otic epithelium around E9.0 by initiating neurogenic gene-mediated programs, such as neurogenin1. These neural precursors generate otic neurons, which are also known as cochleovestibular ganglion (CVG) cells [1]. After CVG complexes are separated into the spiral and vestibular ganglion, developing spiral ganglion neurons (SGNs) promote neuronal outgrowth between E12.5 and E15.5, and regulate peripheral axon guidance to synapse with their target hair cells [2], [3]. This process of auditory neurogenesis depends on well-organized complex signaling networks comprised of trophic factors such as phosphatidylinositol 3 kinase (PI3K)/Akt and insulin-like growth factor I (IGF-I), as well as morphogens, including the Wnt family, cell adhesion molecules and transcriptional regulators [4]–[8]. Several studies of knockout mice and *in vitro* cultures have provided evidence of their important roles in neural survival, neurite outgrowth and nerve innervations to target hair cells of the inner ear [6], [9], [10]. However, spatiotemporal gene expression and the complex molecular networks in neuronal development in the inner ear are not yet fully understood.

Phosphatase and tensin homologue (PTEN), a lipid phosphatase, is negatively regulated by PI3K signaling and contributes to cellular processes including proliferation, differentiation and migration [11]–[14]. Many studies have investigated the function of *Pten* loss in mice, which causes profound alterations in the regulation of cellular maintenance in a cell-type specific manner in various organs [15]–[17]. Recently, we characterized the phenotype of inner-ear-specific *Pten* conditional knockout (cKO) mice, which demonstrated abnormal phenotypes (e.g., ectopic hair cells in the cochlear sensory epithelium and neuronal defects) [15]. In particular, mouse inner ear lacking *Pten* had neuronal deficits such as disorganized nerve fibers with apoptosis of spiral ganglion. Thus, *Pten* is believed to be one of the functional regulators that maintain differentiation of SGNs during inner ear development.

Understanding of the signaling networks during inner ear development may provide molecular information regarding the pathways underlying the maintenance of sensory cells and neurons to prevent hearing impairment. Microarray analysis may provide information that allows prediction of novel signaling networks by analyzing the spatiotemporal pattern of gene expression during inner ear neurogenesis [18]–[20]. Thus, analysis of changes in gene expression profiles and signaling networks obtained from *Pten* mutants may identify potential novel targets and regulatory mechanisms associated with neuronal maintenance during inner ear development. In this study, we explored otic neuron-specific targets of *Pten* signaling to further understand its function in the development of SGNs and the causes of aberrant neural differentiation associated with the *Pten*-deficient inner ear. Our results suggest that secreted phosphoprotein 1 (*Spp1*) and G-protein signaling 4 (*Rgs4*)-mediated networks maintain the neuronal differentiation underlying spiral ganglion development in *Pten*-deficient mice.

## Materials and Methods

### Ethics statement

All mouse procedures were performed according to the guidelines for the use of laboratory animals and were approved by the Institutional Animal Care and Use Committee at Korea Centers for Disease Control and Prevention (KCDC-018-12-1A).

### Tissue dissection and RNA extraction

The generation and characterization of inner ear-specific *Pten* cKO (*Pax2^Cre/+^*; *Pten^loxP/loxP^*) and wild-type (*Pten^loxP/+^* or *Pten^loxP/loxP^*) mice was described previously [15]. *Pten* cKO and littermate wild-type mice were used on E14.5 (60 embryos from each group). The entire inner ear tissues including the cochlea and vestibule, as well as the surrounding otic capsule, were micro-dissected in sterile, chilled phosphate-buffered saline (PBS) under a stereomicroscope (Olympus SZ61, Olympus Corporation, Tokyo, Japan). Three independent pools of inner ear tissues from each group were homogenized with a tissue grinder (Kimble Chase, Vineland, NJ, USA). Total RNA from three independent pools of inner ears was extracted with TRIzol following the manufacturer's instructions (Invitrogen, Carlsbad, CA, USA). To eliminate DNA contamination, total RNA was treated with DNase I (Roche Applied Science, Mannheim, Germany) before use in the microarray analysis or real-time polymerase chain reaction (RT-PCR). The concentration and purity of extracted total RNA were measured using both the spectrophotometric method at 260 and 280 nm, and RNA electrophoresis.

### Microarray data analysis

Gene expression profiles were generated using the Illumina MouseRef-8 version 2.0 Expression BeadChip (Illumina, Inc., San Diego, CA, USA). Three biological replicates (three chips for wild-type samples and three chips for *Pten* cKO samples) were performed for microarray hybridization experiments. Biotinylated cRNA was prepared from 550 ng total RNA using the Illumina TotalPrep RNA Amplification kit (Ambion, Austin, TX, USA). Following fragmentation, 750 ng of cRNA was hybridized to the Illumina MouseRef-8 version 2.0 Expression Beadchip according to the manufacturer's instructions. Array chips were scanned using the Illumina Bead Array Reader Confocal scanner. Microarray data were analyzed using Illumina GenomeStudio Gene expression Module (version 1.5.4) and deposited in NCBI Gene Expression Omnibus Database (GEO, http://www.ncbi.nlm.nih.gov/geo/) (#GSE49562) in agreement with the MIAME requirements. The significance analysis microarrays (SAM) software was used with the false-discovery rate (FDR) set at 0 or 0.05. SAM (FDR = 0) allowed the identification of genes whose expression varied significantly between the wild-type and *Pten* cKO groups [21]. Hierarchical clustering was carried out using the R software [22]. Ingenuity Pathway Analysis (IPA; Ingenuity Systems, http://www.ingenuity.com) tools were used to analyze possible functional relationships between selected differentially expressed genes (DEGs).

### Quantitative reverse-transcription PCR

Quantitative real-time PCR (qRT-PCR) was performed to validate the microarray data. Each pooled RNA sample was converted to cDNA using random hexanucleotide primers with a High Capacity cDNA Reverse Transcription kit according to the manufacturer's instructions (Applied Biosystems, Carlsbad, CA, USA). The list of PCR primer sequences for selected genes is provided in Table S1. 18S rRNA was used as an endogenous control for normalization. The PCR reaction was performed in quadruplicate using SYBR Green PCR Master Mix and an ABI 7500 machine with the version 2.0.6 software under the following conditions (Applied Biosystems): denaturation at 95°C for 10 min followed by 40 cycles of amplification (95°C for 15 sec, 60°C for 1 min). The relative expression level of each target gene in an experimental sample compared with the wild-type sample was analyzed using SDS Relative Quantification (RQ) Manager software as described by the manufacturer (Applied Biosystems). RQ levels were calculated using the comparative C_T_ (2^−ΔΔCT^) method [23]. Relationships between the microarray data and qRT-PCR were analyzed using Pearson's correlation coefficient (*r*) from GraphPad Prism (GraphPad Software, http://www.graphpad.com).

### *In Situ* hybridization

For E14.5 embryos, pregnant mice were sacrificed by decapitation and fixed in 4% paraformaldehyde in PBS overnight at 4°C, dehydrated in 30% sucrose in PBS overnight at 4°C, placed in embedding medium (Tissue Tek OCT compound; Torrance, CA, USA), and stored at −80°C until use. Tissues were sectioned at 10-µm thickness for *in situ* hybridization, which was performed as described previously, with minor modifications [24]. At least three embryos were tested for each selected gene at E14.5. Sense RNA probes were also included as controls, which showed no signal in the inner ear. All primers for RNA probes for otoancorin (*Otoa*), β-tectorin (*Tectb*), parvalbumin (*Pvalb*), *Spp1*, and *Rgs4* are listed in Table S1.

## Results and Discussion

### Identification of genes differentially expressed between wild-type and *Pten* cKO mice at E14.5

**Figure 1:**
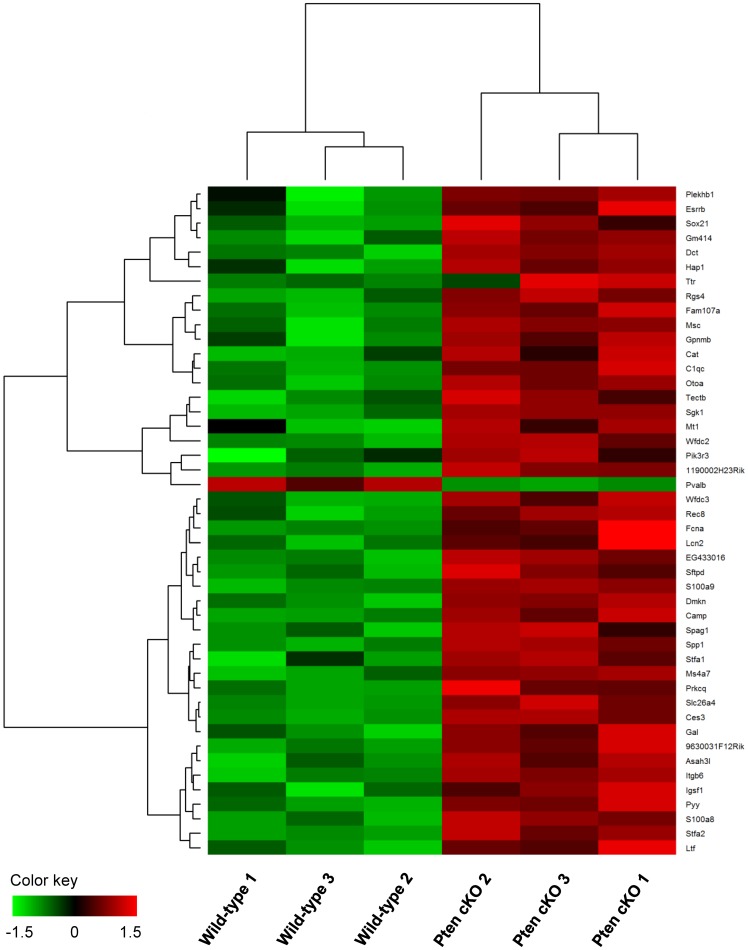
Microarray analysis identifies novel *Pten* targets. Heat maps for relative gene expression of interest (FDR = 0) obtained from three microarrays comparing *Pten* cKO to wild-type embryos. Green and red indicate decreased and increased expression, respectively, in *Pten* cKO mice.

Recently, we reported that *Pten* cKO mice showed severe abnormalities in neuronal maintenance with increased production of hair cells during inner ear development [15]. To identify the changes caused by *Pten* deficiency-induced regulation of genes in the developing inner ear, we analyzed DEGs within inner ears at E14.5. Using SAM analysis, we identified a total of 46 transcripts with an FDR = 0 that significantly distinguished the wild-type and *Pten* cKO groups. Among the transcripts, 45 genes were upregulated and one was downregulated in *Pten* cKO mice, and are listed in Table 1. While the patterns of gene expression between *Pten* cKO and wild-type samples were highly similar according to pair-wise comparisons with correlation coefficients (data not shown), 46 DEGs were significantly selected, and their segregation was clearly shown by clustering analysis of a heat map (Fig. 1).

**Table 1:** Differentially expressed genes in wild-type and *Pten* cKO mice at E14.5.

Target ID	Gene symbol	Definition	Fold change
ILMN_2443330	Ttr	transthyretin	3.94
ILMN_2754364	Ltf	lactotransferrin	2.28
ILMN_2710905	S100a8	S100 calcium binding protein A8 (calgranulin A)	2.00
ILMN_1260585	Stfa2	stefin A2	1.89
ILMN_1259546	Pyy	peptide YY	1.87
ILMN_2803674	S100a9	S100 calcium binding protein A9 (calgranulin B)	1.85
ILMN_2690603	Spp1	secreted phosphoprotein 1	1.83
ILMN_2634484	Tectb	tectorin beta	1.71
ILMN_2988931	Stfa1	stefin A1	1.70
ILMN_2735754	Otoa	otoancorin	1.67
ILMN_2596522	Mt1	metallothionein 1	1.67
ILMN_2712075	Lcn2	lipocalin 2	1.65
ILMN_2805372	Itgb6	integrin beta 6	1.64
ILMN_2648669	Gpnmb	glycoprotein (transmembrane) nmb	1.64
ILMN_1251894	Dct	dopachrome tautomerase	1.57
ILMN_1244081	Rgs4	regulator of G-protein signaling 4	1.56
ILMN_1228497	Esrrb	estrogen related receptor, beta	1.56
ILMN_1244169	Sftpd	surfactant associated protein D	1.52
ILMN_2933022	Plekhb1	pleckstrin homology domain containing, family B (evectins) member 1	1.52
ILMN_1226157	Pik3r3	phosphatidylinositol 3 kinase, regulatory subunit, polypeptide 3 (p55)	1.52
ILMN_1244829	Hap1	huntingtin-associated protein 1	1.51
ILMN_2955694	Spag1	sperm associated antigen 1	1.49
ILMN_2995688	EG433016	predicted gene, EG433016	1.46
ILMN_1213954	Sgk1	serum/glucocorticoid regulated kinase 1	1.45
ILMN_2769777	Msc	musculin	1.45
ILMN_2629112	Asah3l	N-acylsphingosine amidohydrolase 3-like	1.44
ILMN_1258853	Igsf1	immunoglobulin superfamily, member 1, transcript variant 4	1.42
ILMN_2768972	Fam107a	family with sequence similarity 107, member A	1.41
ILMN_2826110	Cat	catalase	1.41
ILMN_2625893	Ces3	carboxylesterase 3	1.40
ILMN_2766604	Camp	cathelicidin antimicrobial peptide	1.40
ILMN_1229131	Wfdc3	WAP four-disulfide core domain 3	1.40
ILMN_2718589	Fcna	ficolin A	1.40
ILMN_1220193	Slc26a4	solute carrier family 26, member 4	1.39
ILMN_2941888	Gm414	gene model 414	1.39
ILMN_2684093	Rec8	REC8 homolog (yeast)	1.38
ILMN_1254295	Sox21	SRY-box containing gene 21	1.38
ILMN_3091003	Ms4a7	membrane-spanning 4-domains, subfamily A, member 7, transcript variant 1	1.37
ILMN_2667829	Prkcq	protein kinase C, theta	1.37
ILMN_2776034	Gal	galanin	1.37
ILMN_2651582	9630031F12Rik	RIKEN cDNA 9630031F12 gene	1.35
ILMN_1229763	Dmkn	dermokine, transcript variant 2	1.34
ILMN_1236758	Wfdc2	WAP four-disulfide core domain 2	1.33
ILMN_2715840	C1qc	complement component 1, q subcomponent, C chain	1.32
ILMN_2593774	1190002H23Rik	RIKEN cDNA 1190002H23 gene	1.31
ILMN_1218223	Pvalb	parvalbumin	−1.62

### Validation of the microarray by quantitative RT-PCR

Among the DEGs, 16 candidate genes were selected to validate by qRT-PCR; the DEGs were chosen for either their fold changes (>1.5) and/or potential roles associated with inner ear development (Table 2). These genes included *Tectb*, *Otoa*, and *Esrrb*, the mutations of which are associated with hearing loss [25]–[30]. In addition, peptide YY (*Pyy*) and integrin beta 6 (*Itgb6*) were identified; these have not been previously reported in the mammalian inner ear. For all analyzed upregulated genes in *Pten* cKO compared to wild-type mice, the average fold change from the qRT-PCR results showed a significant correlation of gene expression changes, as revealed by the microarray data (Pearson's correlation coefficient, *r* = 0.876). This result indicates that changes in the expression of selected DEGs were validated by qRT-PCR while confirming the gene expression results obtained by microarray analysis.

**Table 2:** Genes selected for validation of microarray data by qRT-PCR.

		Average fold change	
Gene	Accession #	Microarray	qRT-PCR
Ttr	NM_013697.3	3.94	15.53
Ltf	NM_008522.3	2.28	5.40
S100a8	NM_013650.2	2.00	6.21
Pyy	NM_145435.1	1.87	4.52
S100a9	NM_009114.1	1.85	7.09
Spp1	NM_009263.1	1.83	3.62
Tectb	NM_009348.3	1.71	6.64
Otoa	NM_139310.1	1.67	3.02
Mt1	NM_013602.2	1.67	4.73
Itgb6	NM_021359.2	1.64	6.42
Dct	NM_010024.2	1.57	3.99
Rgs4	NM_009062.3	1.56	3.24
Esrrb	NM_011934.3	1.56	4.43
Pik3r3	NM_181585.5	1.52	3.58
Hap1	NM_010404.2	1.51	2.58
Pvalb	NM_1218223	−1.62	0.40

### *In situ* expression patterns for selected candidates

To confirm the changes in expression of DEGs in the inner ear, we performed *in situ* hybridization for the selected DEGs, *i*.*e*., *Otoa*, *Tectb*, *Pvalb*, *Spp1*, and *Rgs4* (Figs. S1 and 2). Higher expression of *Otoa* and *Tectb* was observed in the cochlea of *Pten* cKO mice than in the cochlea of wild-type mice (Fig. S1A–D). Many studies have reported that mutations in *Otoa* and *Tectb* cause hearing loss [25], [26], [28]–[30]. Inner ear-specific *Otoa* is reportedly expressed on the surface of the spiral limbus and greater epithelial ridge in the cochlea. Mutant mice lacking *Otoa* showed that otoancorin is required for the attachment of the tectorial membrane (TM) to the surface of the spiral limbus [28], [29]. The TM is composed of collagen proteins, and other non-collagen proteins such as α-tectorin and β-tectorin, and all essential for auditory function. *Tectb*-null mutant mice develop deafness as well as mutation of *Tecta* [30], [31]. Further functional characterization is needed to determine whether a *Pten* deficiency-induced upregulated pattern of *Otoa* and *Tectb* expression leads to abnormal function of the TM.

**Figure 2:**
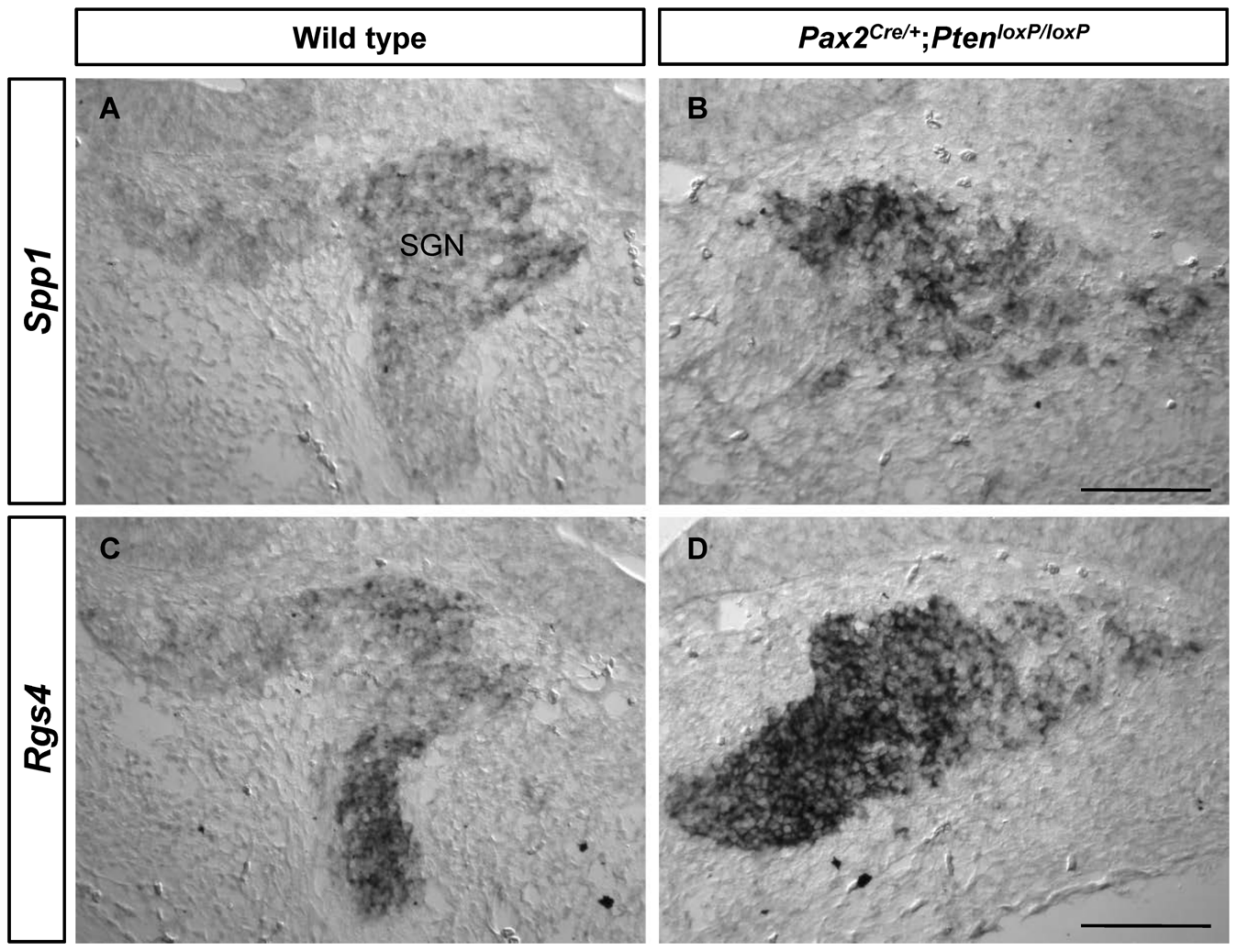
Expression patterns of *Spp1* and *Rgs4* during inner ear development. Expression levels of *Spp1* (A, B) and *Rgs4* (C, D) were examined by *in situ* hybridization at E14.5. Both *Spp1* and *Rgs4* expression were observed in SGNs. Consistent with the microarray results, expression levels of *Spp1* and *Rgs4* were increased in the *Pten* cKO compared to wild-type mice. Scale bars: 100 µm.

In particular, changed expression levels of several genes were detected in the *Pten*-deficient SGNs; i.e., *Pvalb*, *Spp1*, and *Rgs4*. We found that the levels of *Pvalb*, a neuronal marker [32], were downregulated (Fig. S1E, F). Reduced levels of *Pvalb* expression may be explained by the loss of *Pvalb-*expressing neurons in *Pten*-deficient mice. We observed increased levels of *Spp1* (also known as osteopontin, *Opn*) and *Rgs4* expression in *Pten*-deficient SGNs compared to the wild-type (Fig. 2). In the cochlea and vestibular dark cells, *Spp1* may be responsible for regulation of ions in the inner ear fluid. The role of Spp1 in SGNs may be associated with regulation of nitric oxide production, which is considered to be associated with auditory neurotransmission in adenosine triphosphate (ATP)-induced Ca^2+^ signaling [33], [34]. Functionally, several lines of evidence have shown that *Spp1* may play a role in neurodegeneration [35], [36]. Upregulation of SPP1 was detected in lesions or within the cerebral or spinal fluid in patients with neurodegenerative conditions such as Alzheimer's and Parkinson's diseases. *Spp1*-knockout mice showed reduced neurodegeneration induced by MPTP [37]. Following crush injury to the optic nerve, strongly expressed Spp1 by macrophages may have inhibitory effects on axon growth [38]. Therefore, inhibition of axon outgrowth described in *Pten* cKO mice (i.e., shortened length of spiral ganglion toward the modiolus) may be at least partly explained by the dysregulation of *Spp1* expression in SGNs.

Inhibitory regulators of G protein signaling 4 (*RGS4*), a schizophrenia susceptibility gene, is one of the RGS that includes the Gαi/o and Gαq families and is required for modulation of neurotransmission in the nervous system [39], [40]. In mice, the expression of *Rgs4* is observed in peripheral and central neuronal precursors [41], [42]. In the chicken spinal cord, *Rgs4* has been suggested to play a role in neuronal differentiation in cooperation with paired-like homeodomain protein PHOX2b and the basic helix-loop-helix protein MASH1 [41]. Thus, our data suggest that the increased expression of *Rgs4* in the *Pten*-deficient SGNs compared to wild-type mice may play a role in neurogenesis.

### Network analysis

**Figure 3:**
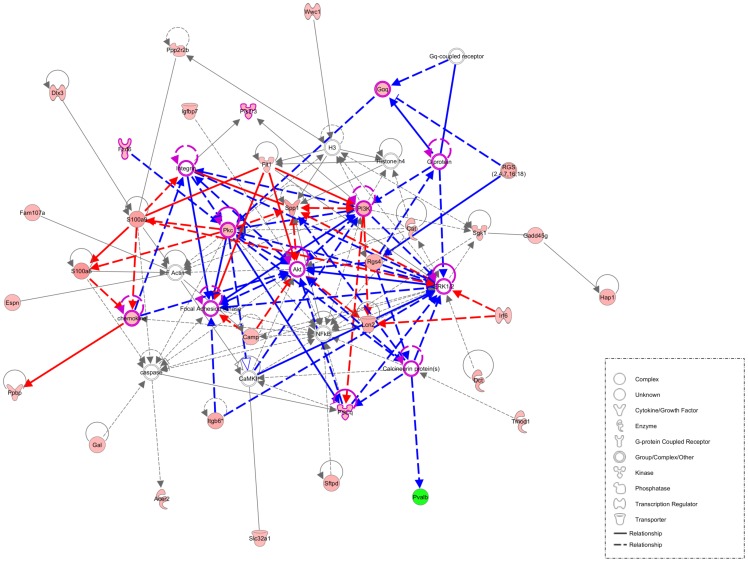
Functional network analysis associated with *Pten*-deficient inner ear. Network analysis using the Ingenuity Pathway Analysis (IPA) software was conducted using selected genes that were differentially expressed and their close relationships. IPA results show two core networks consisted of *Spp1*-(red line) and *Rgs4*-associated interactions (blue line). Genes that were differentially expressed are indicated in pink, and predicted interacting genes (not contained in the microarray data) are indicated in white. Axon guidance signaling pathway-related genes are outlined in magenta. Molecular interactions between connected genes represent direct (solid line) or indirect (dotted line) functional relationships based on the IPA database. Green indicates negative fold changes, while red denotes positive fold changes, according to color intensity.

To examine signaling networks during neuronal maintenance in the *Pten*-deficient inner ear, networks were subjected to IPA analysis with 82 DEGs (FDR<0.05) (Fig. 3). IPA analysis identified significant biological functions, including auditory disease, cell death and survival, and cellular movement (data not shown). Auditory diseases included *Otoa*, *Tectb*, estrogen-related receptor beta (*Esrrb*), and solute carrier family 26 member 4 (*Slc26A4*), which may explain the functional defects of the developing inner ear. Cell death and survival-related genes were enriched, including phosphatase 2A regulatory subunit B beta2 (*Ppp2r2b*), S100 calcium-binding protein A8 (*S100A8*), *S100A9*, insulin-like growth factor-binding protein 7 (*Igfbp7*), and cathelicidin antimicrobial peptide (*Camp*).

In particular, cellular movement included *Spp1*-mediated cell adhesion or migration, which was connected to *S100a8*, *S100a9*, *Integrin*, focal adhesion kinase (*Fak*), lipocalin2 (*Lcn2*), *Camp*, and FMS-related tyrosine kinase 1 (*Flt1*). The chemoattractant activity of SPP1 has been reported in various cell types, some of which interact with integrins such as α_ν_β_3_ [43]–[45]. Dysregulated levels of SPP1 have been implicated in cellular migration; i.e., SPP1 produced by macrophages and microglia induces lateral migration of neuroblasts after focal cerebral ischemia [46]. Furthermore, SPP1 directly induces migration of human lung cancer cells (A549cells) through activation of α_ν_β_3_ integrins, focal adhesion kinase (FAK), p85 subunit of PI3K, serin 473 of AKT and ERK, and the NF-κB-dependent signaling pathway [47]. In our recent study, we detected abnormal neuronal migration with increases in Akt phosphorylation at the Ser473 residue in SGNs of *Pten* cKO mice. Taken together, our results suggest that elevation of *Spp1* produced by SGNs may affect neuronal cell movement in *Pten*-deficient mice compared with wild-type mice. Further experiments are required to elucidate the mechanism by which altered *Spp1* expression induces disturbance of neuronal migration through Akt activation in SGNs.

Regarding the significance of the canonical pathway (data not shown), IPA identified that the Gαq signaling pathway (*p*<0.05) is associated with *Rgs4* (Fig. 3). Gαq signaling is related to axon outgrowth, which is supported by the results from *RGS4* mutant models [48], [49]. Although *Rgs4*-deficient mice exhibit a normal neuronal phenotype, their behavioral abnormality suggests defects in axonogenesis [42]. In zebrafish, an rgs4^−/−^ mutant showed defects in motility and axonogenesis and attenuation of the phosphorylated Akt1 level in the spinal cord [49]. This evidence indicates a novel role for rgs4 in regulating Akt1-mediated axonogenesis. We suggest that increased expression of *Rgs4* in the *Pten*-deficient SGNs, compared with the wild-type, may affect axon outgrowth regulation functionally mediated by the PI3K/Akt signaling pathway due to the increased levels of phosphorylated Akt in SGNs of *Pten* cKO mice. While the biological function of the Rgs4-Akt signaling pathway in the developing SGNs is not fully understood, we suggest that Rgs4-Akt-mediated signaling networks may be associated with neuronal defects in the *Pten*-deficient SGNs (e.g., abnormal path-finding of neurites and irregularly gathered radial bundles).

Finally, IPA analysis revealed two core gene (*Spp1*; red line and *Rgs4*; blue line)-mediated networks in SGNs of the *Pten*-deficient inner ear (Fig. 3). These networks were also associated with the axonal guidance signaling pathway, which includes several mediators, such as G protein, frizzled homolog 6 (*Drosophila*) (*Fzd6*), protein kinase C (*Pkc*), *Akt*, *PI3K*, *Erk1/2*, *Fak*, and *Pkc* theta (*Prkcq*). Therefore, we suggest that partially modulated functions of the axonal guidance signaling pathway are involved in axonal development in *Pten* cKO mice [50]–[53].

## Conclusions

In this study, we investigated profiles of significantly differentially expressed transcripts and their respective networks associated with *Pten* deficiency in the developing inner ear at E14.5. We suggest the presence of core signaling networks mediated by upregulated expression of *Spp1* and *Rgs4*, which also include several key factors associated with apoptosis, cellular movement, and axon guidance. This may be explained in terms of phenotypic defects implicated in neuronal differentiation of *Pten*-deficient SGNs during inner ear development (e.g., neuronal apoptosis, shortened axon length, abnormal cell movement, and irregular neurite path-finding of SGNs). Our gene expression profiles will facilitate understanding of the neuronal maintenance in developing spiral ganglion. However, the functional roles of these candidates should be examined in future studies.

## Supporting Information

Figure S1 Expression patterns of *Otoa*, *Tectb*, and *Pvalb* during inner ear development at E14.5. Expression levels of *Otoa* (A, B), *Tectb* (C, D), and *Pvalb* (E, F) were determined by *in situ* hybridization at E14.5. *Otoa* transcripts were identified on the surface of the spiral limbus and greater epithelial ridge in the cochlea (A, B). Expression domains of *Tectb* were observed in the sensory epithelium of the cochlea (C, D). The neuronal marker *Pvalb* was expressed in SGNs (E, F). Consistent with the microarray data, the expression levels of *Otoa* (B) and *Tectb* (D) were higher, and that of *Pvalb* (F) was lower, in *Pten* cKO mice than in wild-type mice. Scale bars: 100 µm.
(TIF)

Table S1 Primer sets for qRT-PCR and *in situ* hybridization probe.
(DOCX)
