# Supplementary figures and images for: Patterns of Gene Expression Associated with Pten Deficiency in the Developing Inner Ear
Source: PLoS One. 2014 Jun 3;9(6):e97544. doi: 10.1371/journal.pone.0097544 (PMC4043736; doi:10.1371/journal.pone.0097544)

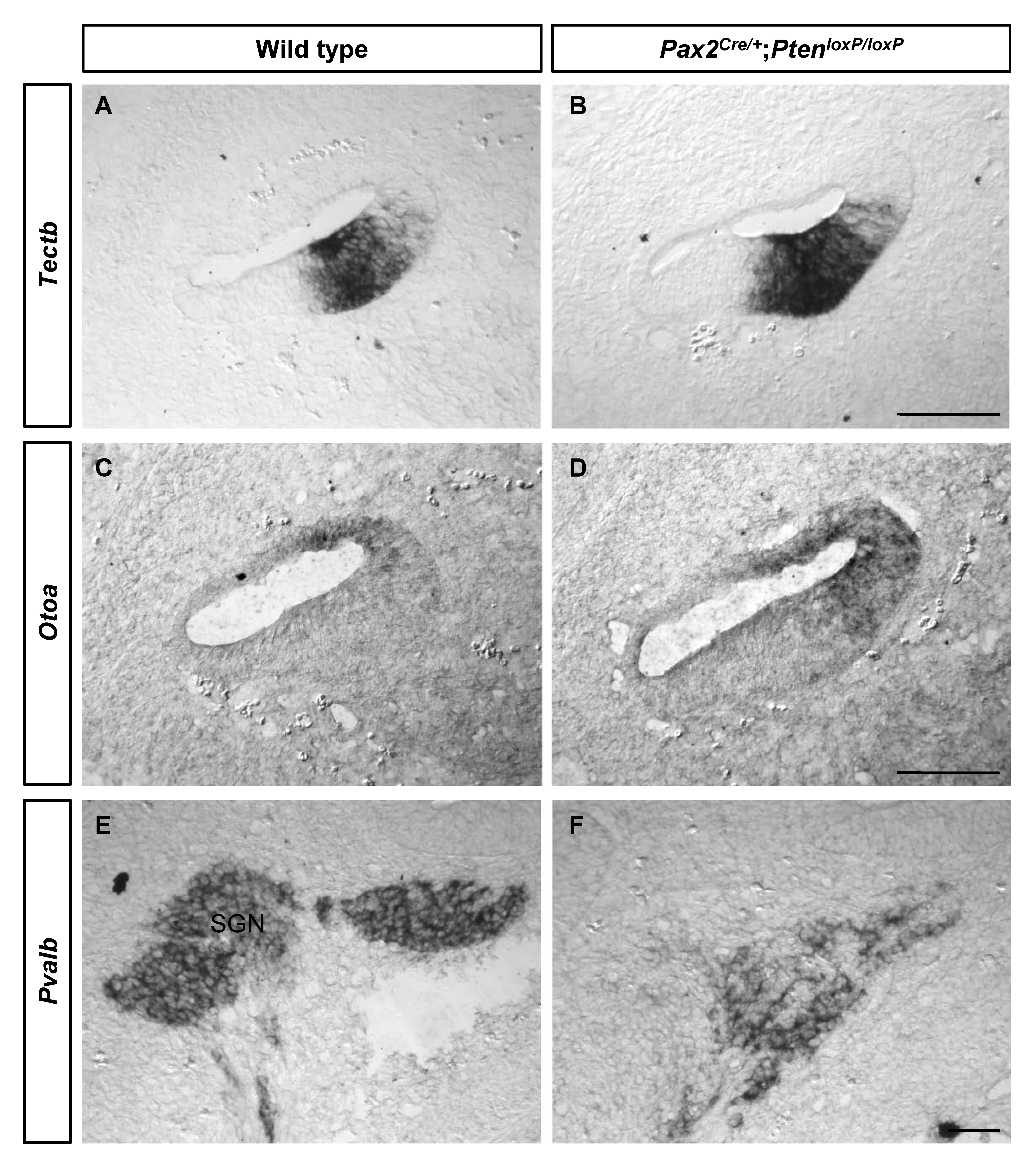

Supplement: Figure S1 — Expression patterns of Otoa, Tectb, and Pvalb during inner ear development at E14.5. Expression levels of Otoa (A, B), Tectb (C, D), and Pvalb (E, F) were determined by in situ hybridization at E14.5. Otoa transcripts were identified on the surface of the spiral limbus and greater epithelial ridge in the cochlea (A, B). Expression domains of Tectb were observed in the sensory epithelium of the cochlea (C, D). The neuronal marker Pvalb was expressed in SGNs (E, F). Consistent with the microarray data, the expression levels of Otoa (B) and Tectb (D) were higher, and that of Pvalb (F) was lower, in Pten cKO mice than in wild-type mice. Scale bars: 100 µm. (TIF) [file pone.0097544.s001.tif]
