# Supplementary material for: Patterns of Gene Expression Associated with Pten Deficiency in the Developing Inner Ear
Source: PLoS One. 2014 Jun 3;9(6):e97544. doi: 10.1371/journal.pone.0097544 (PMC4043736; doi:10.1371/journal.pone.0097544)
Supplement: Table S1 — Primer sets for qRT-PCR and in situ hybridization probe. (DOCX) [file pone.0097544.s002.docx]

Table S1. Primer sets for qRT-PCR and *in situ* hybridization probe.

| **Experiment** | **Target gene** | **Sequence** |  |
| --- | --- | --- | --- |
| qRT-PCR | Dct | F:5'-GGT TGC TGG CTT TTC TTC AA-3' | R:5'-GGC TTC CTC CGT GTA TCT CTT-3' |
|  | Esrrb | F:5'-TTT TTA ACC GGC AGT CCT TC-3' | R:5'-GAG ATA ATC CCA GGG CAT GT-3' |
|  | Hap1 | F:5'-ACA TCC GTG CTC ACA CAG AG-3' | R:5'-CAT CAA TAC AAA GGC TGA CTA GC-3' |
|  | Itgb6 | F:5'-TTG GAG TCT GGA GCA ACC A -3' | R:5'-ATT TCA GCA ATA ACA CAA AAA CAG C-3' |
|  | Ltf | F:5'-CCA TCT GGT CTC CTC ACT CC-3' | R:5'-CAC CTC ATA TGA CGA GAA ACT CA-3' |
|  | Mt1 | F:5'-TTC CTA TAC AGT TCC ACC CTG TT-3' | R:5'-TCA AAC AGG CTT TTA TTA TTC ACG-3' |
|  | Otoa | F:5'-GTA CCC TGC TGC TTC TGA CG-3' | R:5'-TGA CTC TGC ACA CCT TCA CC-3' |
|  | Pik3r3 | F:5'-TCC ATT CTT GGT CCT TTG CT-3' | R:5'-AGG GTG GTT GTC ACA AAT GG-3' |
|  | Pvalb | F:5'-TTA CTC CCC CAT CCT CTA TGG-3' | R:5'-GTC CGA TTG GTA CAG CCT TT-3' |
|  | Pyy | F:5'-ACC GAC TTC ACT TGC ATG TTT-3' | R:5'-AAC ACA CAC AGC CCT CCA GT-3' |
|  | Rgs4 | F:5'-CAT GAA CTG CAA AGC CCT CT-3' | R:5'-TGG TCT CTG CTA GCC TTG GT-3' |
|  | Rn18S | F:5'-CCG CAG CTA GGA ATA ATG GA-3' | R:5'-CCC TCT TAA TCA TGG CCT CA-3' |
|  | S100a8 | F:5'-ATG CCG TCT GAA CTG GAG AA-3' | R:5'-TTC CTT GTA TAT TGG AAT AAT TGT GG-3' |
|  | S100a9 | F:5'-GCA AAG GCT GTG GGA AGT AA-3' | R:5'-GGC CAT TGA GTA AGC CAT TC-3' |
|  | Spp1 | F:5'-TGG GCT CTT AGC TTA GTC TGT TG-3' | R:5'-CAG AAG CAA AGT GCA GAA GC-3' |
|  | Tectb | F:5'-TGT TTA GCC GCA GGC TAG TG-3' | R:5'-GAT TGG AGC ATG AAC TAG ATA CCA-3' |
|  | Ttr | F:5'-CAA AGC AGT AGC ATC CCA TTT-3' | R:5'-AGA ATG CTT CAC GGC ATC TT-3' |
| *In situ* hybridization probe synthesis | Otoa | F:5'-GGA CAC CCT TCA ACG TCA CT-3' | R:5'-GAC AGC ACC TTT TCC TGA GC-3' |
|  | Pvalb | F:5'-TTC TGA AGG GCT TCT CCT CA-3' | R:5'-TGC AGA GAT TGA ACG AGG TG-3' |
|  | Rgs4 | F:5'-GCT AAG GGG TGA GCA CTC TG-3' | R:5'-TGG TCT CTG CTA GCC TTG GT-3' |
|  | Spp1 | F:5'-TGG CAG CTC AGA GGA GAA GCT-3' | R:5'-GGC ATG CTC AGA AGC TGG GCA-3' |
|  | Tectb | F:5'-CTG TTT GCG GGA GTA GAA GC-3' | R:5'-CAT ATT TGG CAA AGG GCT GT-3' |
